# Advantages and disadvantages of online psychotherapy and decisions to use it in the era of the COVID-19 pandemic - analysis of mediation variables

**DOI:** 10.3389/fpsyt.2025.1679186

**Published:** 2025-12-02

**Authors:** Joanna Furmańska, Emilia Rutkowska, Håkan Lane, Johannes Meixner, Cristiana C. Marques, Maria João Martins

**Affiliations:** 1 Department of Clinical Psychology and Psychoprophylaxis, Institute of Psychology, University of Szczecin, Szczecin, Poland; 2 Research Group Scientific Methods, Brandenburg Medical School, Fontanestadt Neuruppin, Germany; 3 Department of Psychiatry and Psychotherapy, Brandenburg Medical School, Immanuel Klinik Rüdersdorf, Rüdersdorf, Germany; 4 HEI-Lab: Digital Human-Environment Interaction Labs, Lusófona University, Lisbon, Portugal; 5 Center for Research in Neuropsychology and Cognitive and Behavioural Intervention (CINEICC), Faculty of Psychology and Educational Sciences, University of Coimbra, Coimbra, Portugal; 6 University of Coimbra Health Services, University of Coimbra, Coimbra, Portugal

**Keywords:** online therapy, psychotherapists, cognitive factors, psychological factors, epidemiological and institutional factors, decisions, COVID-19

## Abstract

**Introduction:**

In recent years, online psychotherapy has gained importance as a flexible, cost-effective and widely available alternative to face-to-face therapy. Existing research suggests that psychotherapists see both the advantages and potential limitations of this form of work. It can be assumed that the perceived advantages and disadvantages of online therapy constitute the primary direct factors influencing decisions to adopt this form. However, in the era of the pandemic, it seems important to assess to what extent emotional factors - such as fear of infection or pandemic-related fatigue - and institutional factors - such as perceived infection rates or trust in the official recommendations, mediate this relationship. Therefore, this study aimed to examine how cognitive evaluations, accompanying emotions, and institutional conditions influenced psychotherapists’ decisions to adopt remote practice during the COVID-19 pandemic.

**Material and methods:**

The study involved 283 psychotherapists from four European countries: Poland, Portugal, Germany and Sweden. An original interview created for the purposes of the study was used, to collect sociodemographic data related to the professional functioning of psychotherapists and aimed at learning about the advantages and disadvantages of online therapy in the opinion of psychotherapists. In addition, the Hospital Anxiety and Depression Scale (HADS), the Fear of Contracting COVID-19 Scale (FCS Covid-19), and the Pandemic Fatigue Scale (PFS) were used.

**Results:**

The results showed that the vast majority of therapists recognized both advantages (accessibility and convenience, health protection, flexibility, cost reduction and practicality, comfort and familiarity with the environment and functionality in online diagnosis and therapy) and disadvantages (problems with effectiveness, technical, safety and organizational difficulties) of online therapy. The analysis of the decision-making process showed no direct relationship between the perception of the advantages and disadvantages of online therapy and the decision to use it. Mediation analyses showed no indirect effects for advantages; for disadvantages, two institutional mediators operated in opposite directions (government recommendations: positive; society recommendations: negative), while emotional mediators were non-significant. This means that government recommendations and recommendations of psychotherapeutic societies mediate the relationship between perceived limitations and the decision to use online therapy.

**Discussion:**

The results obtained make a significant contribution to the understanding of the advantages and limitations of online therapy perceived by psychotherapists and the factors directly and indirectly related to the decision to use it.

## 1 Introduction

Online psychotherapy, while not a commonly selected form of therapy prior to the pandemic (1–3), during and after the lockdowns, it has gained importance as a flexible, cost-effective and widely accessible option (4–7) during and after the lockdowns. Research shows that this form of therapy was utilized by patients across all age cohorts, from adolescents to older adults, and was adopted by therapists in response to insurance recommendations and individuals risk assessments (8–11). It seems that therapists’ perceptions of the benefits and limitations of online psychotherapy significantly influenced their decision to see patients in person or provide remote treatment.

### 1.1 Advantages and limitations of online therapy – a synthesis of the findings so far

The main advantages of online psychotherapy, described in the literature, include, among others: the ability to schedule sessions at any time – for example: in the evening or on weekends, elimination of travel requirements – sessions can be conducted from any location with internet access, reduced costs associated with travel and the therapy fees, a broader selection of therapists, including those based in other cities or countries, enhanced discretion and reduced stigma concerns, as well as the ability to conduct couple or family therapy even when members are geographically dispersed (9, 12, 13).

Nevertheless, despite the benefits and opportunities offered by online psychotherapy, psychotherapists and researchers point out the limitations of this form of work, among others: reduced communication quality or session disruptions due to unstable internet connections or inadequate equipment on any site either therapists and clients (11, 14, 15); concerns regarding privacy and session confidentiality (9, 13, 16), challenges in interpreting nonverbal cues – reduced image quality and limited camera frames impede observation of facial expressions, gestures and posture, complicating emotional assessment and intervention selection (11, 14). In addition, therapists report the inability to employ “physical” tools, such as sticky notes, play therapy toys or art materials (13, 14). Many clients also report screen fatigue and perceived relational distance, which may diminish the authenticity of the therapeutic connection (17–19). Finally, there are ethical dilemmas concerning recording storage, data protection and the therapist’s duty of care when clients are located in jurisdictions different from the therapist’s practice (12). These analyses demonstrate that psychotherapists recognize the benefits of online therapy, but also acknowledge the limitations (20).

### 1.2 Context of the COVID-19 pandemic and decisions regarding the adoption of online therapy

In the face of the COVID-19 pandemic, the risk of SARS-CoV-2 infection, and recommendations issued by health authorities and professional bodies, including psychotherapeutic societies, therapists faced the decision: to either continue working in-person practice in their offices or transition to a remote form.

The question arises whether the choice of online therapy was predominantly driven by a deliberative evaluation of its advantages and disadvantages, or whether – in pandemic conditions – it was driven by emotional factors - such as fear of infection or the need for a sense of security, and epidemiological and institutional factors, related, e.g. to the subjective perception of the infections surges or trust in official recommendations, that mediated this decision.

It was assumed that perceived advantages — e.g., flexibility, reduced costs, broader accessibility — and disadvantages — e.g., technical challenges, limited nonverbal communication — are the primary direct influences on therapists’ decisions to adopt this form of work. These assumptions can be applied to expected utility theory (21) and the theory of planned behavior (22), where rational assessments of costs and benefits determine intentions and behaviors.

On the other hand affective heuristics (23) and Dual-Process Models (24) highlight the role of emotions – fear of infection (25) and pandemic fatigue (26) – as factors that filter and modify rational calculations. This means that strong emotions, for example, may reinforce protective behaviors — such as the shift to remote work — or hinder the adoption of novel therapeutic forms. In addition, the subjective perceptions of (the harmfulness of) infection waves or the trust in government recommendations shapes both cognitive evaluations and the intensity of emotional reactions. For example research has shown that in regions or periods with greater threat or low institutional trust, therapists may have experienced heightened fear, which in turn may have altered how perceived benefits and drawbacks influence the decision to provide online therapy) (27).

Therefore, in accordance with the above, during the pandemic, emotional and institutional - epidemiological factors likely mediated therapists’ evaluations of benefits and drawbacks, – a higher level of epidemiological threat or low trust in institutions may prompt the selection of online therapy despite its inherent limitations.

### 1.3 Knowledge gap and the purpose of current study

So far, few studies have directly compared these approaches in psychotherapists’ decisions to adopt online therapy. A review of the reveals a gap of knowledge: no comprehensive research has simultaneously (A) assessed how cognitive evaluations of the benefits and drawbacks of online therapy have influenced its adoption, (B) examined emotional (e.g., fear of infection, pandemic fatigue) and institutional (e.g., infection waves, trust in guidelines, health status) mediators of that influence, and (C) integrated qualitative insights from therapists with quantitative mediation analyses. Article fills this gap with a dedicated focus on the time period of the COVID-19 pandemic.

The aim of this study is to address this gap by determining how cognitive evaluations, emotional responses, and institutional conditions affected psychotherapists’ decisions to use online therapy during the COVID-19 pandemic. The current study involves an analysis of psychotherapists’ decision-making during the pandemic through a mixed-methods design: a qualitative analysis (interviews exploring individual motivations and reservations) followed by a quantitative analysis (mediation modeling) to elucidate how cognitive evaluations, emotional factors, and institutional conditions jointly shaped the adoption of online therapy.

In our study, we assumed that the primary impetus for selecting online therapy is a conscious and rational evaluation of its benefits and costs. In our model, the perception of pros and cons is therefore the first, direct predictor of the decision to use online therapy (cf. 21, 22). Additionally, we assume that under extreme pandemic stress, emotional factors — e.g., fear of SARS-CoV-2 infection (25) and pandemic fatigue (26) — mediate between benefit – cost appraisals and the ultimate decision to adopt online therapy. As a second set of mediators, we also included epidemiological and institutional factors — such as local infection rates and trust in official guidelines, given their influence on cognitive evaluations and emotional responses.

The entire framework as Dual-Process Models (24), in which cognitive evaluations (System 2) and rapid emotional responses (System 1) operate in tandem. The former serves as the primary driver of choice, while the latter acts as a filter that modulates the magnitude and direction of rational influences. Institutional factors constitute a third contextual layer, mediating and modulating these processes.

We determined the directionality of these relationships based on the Technology Acceptance Model (TAM) and the Unified Theory of Acceptance and Use of Technology (UTAUT), where perceived usefulness and effort expectancy (i.e., benefits and costs) directly predict behavioral intention.

Secondly, during the pandemic, emotional responses and institutional context functioned as filters mediating these evaluations – hence casting them as mediators enables us to understand how therapists’ appraisals of remote therapy’s pros and cons translated into a choice of practice mode.

With this structure, we can not only examine the direct relationship between the pros and cons of online therapy and the decision to use it, but also assess the extent to which emotional and contextual factors influence this relationship.

## 2 Material and methods

### 2.1 Research model

The study employed an exploratory sequential mixed-methods design, conducting the qualitative phase prior to the quantitative phase. This model is based on the exploratory sequential model outlined by Creswell and Plano Clark (28). The qualitative phase, grounded in social constructivism, aims to identify the categories psychotherapists use to conceptualize the benefits and drawbacks of online therapy. The primary research questions are: (A) Do psychotherapists see the advantages and disadvantages of online therapy? (B) What advantages and disadvantages of online therapy do psychotherapists report?

The quantitative phase grounded in post-positivism, facilitates standardization, generalization of qualitative findings, and quantification of relationships among variables. This permits empirical testing of hypotheses regarding the relations between cognitive factors (reported advantages and disadvantages of online therapy) and therapists’ decisions to conduct online sessions. In the quantitative analysis, it was decided to check how the cognitive factor is related to the decision to use this form of work, as well as to identify potential emotional mediators (fear of contracting COVID-19, pandemic fatigue, general anxiety and depression) and epidemiological and institutional mediators (the motives indicated by psychotherapists for the decision to conduct online therapy, i.e. epidemiological situation, government recommendations, recommendations of psychotherapeutic associations, the patient’s health condition and the therapist’s health condition), which may mediate the relationship between the perception of the advantages and disadvantages of online therapy and the decision to use this form of work.

The main research questions the current analyses answer are: (C) Is the cognitive factor in the form of perceiving the advantages and disadvantages of online therapy related to the actual decision to use online therapy? (D) Do emotional factors, such as fear of contracting COVID-19, pandemic fatigue, general anxiety and depression, mediate (mediate) the relationship between cognitive assessment (assessing the pros and cons of online therapy) and the decision to use this form of therapy? (E) Do epidemiological and institutional factors, such as the epidemiological situation, government recommendations, recommendations of psychotherapeutic societies, the patient’s health condition and the therapist’s health condition, mediate the relationship between cognitive assessment and the decision to use this form of therapy?

The combination of these two approaches will allow for a more complete understanding of the phenomenon than using only a fragmentary perspective. The adopted research model has an exploratory character; therefore, we do not formulate hypotheses but focus on research questions, which is consistent with the approach recommended in situations where the studied phenomenon is new and has not yet been sufficiently examined (29).

### 2.2 Measures

#### 2.2.1 Original interview

For this study, an interview questionnaire was created, which included closed questions concerning sociodemographic data (e.g. country, gender, age) and related to professional functioning (e.g. therapeutic approach, form of work, number of working hours), as well as open questions, designed to explore the advantages and disadvantages of online therapy. The open questions were structured as follows: A) Describe what the benefits/advantages of online psychotherapy (give some example); B) Describe what drawbacks you see in online psychotherapy (give some example)?

#### 2.2.2 Hospital Anxiety and Depression Scale

The questionnaire (30) consists of 14 items, seven questions relating to anxiety (HADS-A) and seven questions for depression (HADS-D). It is a self-report scale which focuses on non-physical symptoms. The literature indicates Cronbach’s Alpha score of HADS–A α = .83 and HADS–D α = .82, while in this study HADS–A α = .85 and HADS–D α = .80.

#### 2.2.3 Fear of Contracting COVID-19 Scale

FCS COVID-19 (31) is a 7-item scale. It is based on the AIDS Fear Scale (32) but was specifically developed to assess fears related to COVID-19. Respondents indicate the level of fear and anxiety they feel in connection with situations related to a possible infection. In the literature, α = 0.91 was obtained, while in this study the result was α = 0.89.

#### 2.2.4 Pandemic Fatigue Scale

The PFS (33) consists of 6 items and is a valid and cost-effective measure to assess pandemic fatigue. Pandemic fatigue is understood as a component of separate but strongly correlated factors such as information fatigue (IF) and behavioral fatigue (BF), which contribute to the overall feeling of pandemic fatigue. In original study a Cronbach’s alpha score of.82/.86 for the IF factor,.72/.79 for the BF factor, and.83/.88 for the full PFS. In this study Cronbach’s alpha score 0.81 for the Information Fatigue (IF) factor, 0.76 for the Behavioral Fatigue (BF) factor and 0.84 for the full PFS.

In this study, both the internal and external validity of the tools used were analyzed and taken into account.

### 2.3 Procedure, participants, and recruitment

We recruited actively practicing psychotherapists and their selection was based on a pilot phase and a main phase. In the first phase, short-term snowball sampling was used within the authors’ environment. In the second, main phase, purposeful sampling with elements of geographic stratification was employed, meaning that official membership lists of psychotherapy societies and associations were used, which served as the basis for sending invitations to participate in the study. Furthermore, public contacts to public psychotherapy institutions and private practices were also used in each state or province, and invitations were posted in specialized Facebook groups for psychotherapists. A request to participate in the study was sent at most twice. The survey was conducted in four European countries, namely Poland (Eastern Europe), Portugal (Southern Europe), Germany (Central Europe) and Sweden (Northern Europe).

The inclusion criteria was having completed or currently enrolled in a postgraduate psychotherapy program [4–5 years] and/or holding a certification and practicing an evidence-based modality (e.g. cognitive-behavioral, integrative, psychodynamic or psychoanalytic, systemic, existential and Gestalt, Ericksonian therapy). All participants were required to provide informed consent to participate in the study prior to the study.

Exclusion criteria were not having professional experience as psychotherapists, lack of informed consent to participate in the study, as well as incomplete surveys.

The ethics committee’s approval for the study was issued by XXXX (data hidden so as not to interfere with the review process) prior to data collection. Participation in the study was completely voluntary, and it was possible to withdraw from participation at any time, without any consequences. Data collection began in February 2022 and ended in March 2022.

#### 2.3.1 Descriptive characteristics of participants

The study involved 283 psychotherapists. The mean age of the participants was 45.9 years (SD = 12.0), ranging from 23 to 80 years. The average work experience was 14.4 years (SD = 9.6; range: 1–45 years). The majority of respondents were women (78.4%), men accounted for 21.2%, and non-binary individuals – 0.4%. The participants came from Poland (31.8%), Portugal (27.6%), Germany (23.3%) and Sweden (17.3%).

In terms of education, the majority of psychotherapists held a degree in psychology (77.0%), and the rest held degrees in pedagogy (6.0%), medicine (7.1%), sociology (2.5%), or other disciplines (7.4%). Most of the respondents had a psychotherapist certificate (44.5%) or had completed a 4–5-year psychotherapy course (30.4%), and 25.1% were undergoing such training.

The most frequently reported therapeutic approaches were: psychodynamic and psychoanalytic (27.2%), integrative (20.1%) and cognitive-behavioral (19.8%). A smaller percentage of therapists indicated a systemic approach (17.2%), existential and Gestalt (7.1%), Ericksonian therapy (3.0%) and other (5,6%).

The majority of psychotherapists worked in large cities with more than 100,000 inhabitants (73.5%), 25.4% in small towns (1,000–100,000), and 1.1% in small villages (less than 1,000 inhabitants). The vast majority worked exclusively in private practice (60.3%), 29.1% combined work in the private and public sectors, 6.0% worked only in the public sector, and 4.6% indicated other forms of employment.

Psychotherapists reported an average of 6.7 hours of online work per week (SD = 8.1; range: 0–40) and a total of 22.3 hours of therapeutic work per week (SD = 11.7; range: 8–55). Most of the therapists surveyed (86.6%) worked remotely, while the remaining 13.4% did not use remote sessions.

### 2.4 Statistical analysis

Qualitative data obtained from the open-ended interview questions were subjected to content analysis using a categorical approach. The procedure involved identifying the main areas of meaning in the participants’ statements, assigning them to categories and subcategories, and calculating the frequency of occurrence of individual topics. The analysis process was based on a semantic approach (34–36), focusing on the overt meaning of the statement. The analysis was inductive – the categories were derived from the data, without prior theoretical assumptions. The analysis was conducted according to Braun and Clarke’s (34) thematic analysis procedure, which involved coding participants’ responses, grouping them into categories, and identifying overarching themes. The coding agreement between the two independent encoders was assessed using the Cohen’s κ coefficient (κ = 0.83), which indicates the high reliability of the analysis. Following the approach described by Creswell and Plano Clark (28), we took steps to ensure reliability and consistency of the analysis. Two investigators participated in the coding process, and inter-rater reliability was ensured by jointly discussing and reconciling codes until consensus was reached, indicating that the coding criteria were clearly defined and objective. To enhance transparency and structure the results, elements of a quantitative approach were used – the frequency (N) and percentage (%) of statements assigned to a given category were counted. This approach allowed us to first distinguish the main themes, and second, present the dominant and marginal topics.

In the quantitative analysis, a set of statistical tests was conducted, such as tests of dependency, as well as predictive and mediation analyses. In the first step, chi-square independence tests were used to assess the relationship between dichotomous variables, i.e. perceiving the advantages or disadvantages of online therapy and the decision to conduct it (0 = no, 1 = yes). In the second step, logistic regression was used to investigate the predictive value of the perceived advantages and disadvantages of online therapy in relation to the decision to provide it. In addition, to increase the precision of interpretation, a set of univariate logistic regressions was also performed, in which the decision to use online therapy was predicted by individual advantages and disadvantages identified in the previous qualitative analysis. In order to investigate potential mediating effects, a mediation analysis using a bootstrapping procedure (2,000 resamples) was carried out as recommended by Hayes (37). We analyzed whether selected psychological variables (e.g. fear of COVID-19, symptoms of depression and anxiety, pandemic fatigue) and epidemiological and institutional variables (e.g. government or professional recommendations, health status of the therapist or patient) mediate the relationship between the perception of the advantages or disadvantages of online therapy and the decision to use it. Indirect effects (a·b) and their significance were assessed on the basis of 95% confidence intervals. An effect was considered significant if the confidence interval did not contain zero.

Since our goal was to examine direct and indirect relationships between variables—perceived advantages and disadvantages of online therapy, emotional factors, and the decision to use this form of therapy—we selected logistic and linear regression models and bootstrapping mediation analysis for statistical analyses. This is because regression models are a well-established method in psychological research for testing such relationships with observable variables and allow for direct interpretation of the strength and direction of effects. We also considered that the basic psychometric properties of the selected instruments (e.g., factor structure, validity, and reliability) had already been confirmed in validation studies.

## 3 Results

In accordance with the adopted research model and research questions, the qualitative analysis is presented first, i.e. an analysis of the advantages and disadvantages of online therapy perceived by psychotherapists. In the next part, the quantitative analysis is presented in two stages: first, the advantages of online therapy, followed by its disadvantages, each examined in the context of all analyzed variables and their interrelationships.

### 3.1 Qual–quant integration

Categories identified in the qualitative phase (access/continuity, flexibility, technical barriers, confidentiality, therapeutic alliance/diagnostic constraints) informed the construction of binary predictors (advantages/disadvantages) and the selection of candidate mediators. Institutional reasons frequently cited by therapists in interviews (e.g., government and professional-society guidance) were therefore modeled as epidemiological–institutional mediators in the quantitative phase.

### 3.2 Advantages and disadvantages of online therapy in the perception of psychotherapists - qualitative analysis

The results of the study on the perception of the advantages and disadvantages of online therapy are presented in **Table 1**. The vast majority of respondents reported both advantages (91.55%) and disadvantages (87.30%) of this form of work. This finding indicate that psychotherapists acknowledge both the benefits and limitations of online therapy.

**Table 1:** Perception of the advantages and disadvantages of online psychotherapy by psychotherapists (N = 283).

Advantages of online therapy		Disadvantages of online therapy	
Yes	No	Yes	No
259	24	242	36
91.52%	8.48%	87.10%	12.90%

#### 3.2.1 The advantages of online therapy noticed by psychotherapists

A qualitative analysis of the obtained data allowed for a description of the categories of advantages of online psychotherapy mentioned by psychotherapists. **Table 2** presents the main categories, subcategories and examples of descriptions and frequency of perceiving a given advantage of online therapy, which were identified as part of the qualitative analysis based on the statements of psychotherapists.

**Table 2:** Advantages of online therapy noticed by psychotherapists.

Main category	Subcategory	Description	N	%
1. Accessibility and Convenience	1.1. Geographical flexibility	Ability to participate from anywhere	107	37.80%
	1.2. Continuity of care	Possibility of therapy despite changing location	72	25.44%
	1.3. Time saving	No travel saves time	53	18.72%
2. Health Security	2.1. Protection against infection	Reducing the risk of infection due to the lack of physical contact	45	15.90%
3. Flexibility	3.1. Flexibility in Schedule	Adjusting the time of the session to individual needs	28	9.89%
	3.2. Adaptation to Crisis Situations	Ability to conduct sessions despite sudden events	4	1.41%
	3.3. Possibility of Sessions at Unusual Times	Possibility to work non-standard hours	1	0.35%
4. Cost Reduction and Practicality	4.1. Cost Efficiency	Eliminate commuting costs	10	3.53%
	4.2. Minimum organisational requirements	No need to rent a practice, simplicity of organization	7	2.47%
5. Comfort and Knowledge of the Environment	5.1. Patient Comfort at Home	The patient feels more at ease in their own environment	23	8.12%
	5.2. Sense of Security at Home	Familiar surroundings strengthen the sense of security	11	3.88%
6. Functionality in online diagnosis and therapy	6.1. Insight into the Patient’s Environment	Ability to observe the patient’s surroundings in the natural environment	17	6.00%
	6.2. Session structure	Easier planning and predictability	10	3.53%
	6.3. Possibility of providing psychological education	Ease of use of digital materials in the session	4	1.41%

##### 3.2.1.1 Accessibility and convenience

The first group of advantages of online psychotherapy, indicated by psychotherapists, was the availability and convenience of this form of psychotherapy. As many as 44.16% of all respondents indicated that this is a significant advantage of remote therapy. This category includes two key aspects: continuity of therapeutic care – i.e. the ability to continue therapy regardless of location, and time savings by eliminating the need to travel.

Continuity of therapeutic care, understood as the possibility of conducting therapy despite the change of the patient’s or therapist’s place of residence, was indicated as an advantage by as many as 25.44% of the respondents. Qualitative analysis of psychotherapists’ statements indicates that it a) is understood as the possibility of continuing therapy regardless of the patient’s or therapist’s location; b) increases the stability of the psychotherapeutic process; and c) reduces the number of interruptions in contact. There were numerous statements indicating the possibility of conducting sessions with patients who have moved, live abroad or are temporarily away from their place of residence. Psychotherapists also drew attention to the possibility of working with people who - for health or organizational reasons - could not benefit from inpatient therapy, e.g. people with disabilities, young mothers or patients from rural areas. Online psychotherapy also made it possible to maintain a therapeutic relationship in the event of illness or in the conditions of a pandemic.

Time savings, referring to the lack of the need to travel to sessions, which allows for more effective time management, were indicated as an advantage of online psychotherapy by 18.7% of respondents. These results highlight that psychotherapists highly value the organizational and logistical facilities provided by remote therapy. Analysis of the obtained statements indicates that therapists noticed that their patients no longer had to waste time on the move, which makes therapy more accessible, especially in conditions of busy schedules and other life commitments.

##### 3.2.1.2 Health security

The second group of advantages of online psychotherapy included health security, and more specifically protection against infection, which was indicated as an advantage of online psychotherapy by 15.9% respondents. These statements referred primarily to reducing the risk of infection due to the lack of physical contact during the session. This result shows that for a significant proportion of psychotherapists, the health factor, especially in the context of the pandemic, was a real and appreciated benefit of remote work. The benefits of the possibility of therapeutic work in isolation or quarantine conditions, without the need to interrupt the therapeutic process, were also pointed out. In addition, online therapy was also seen as more comfortable and free, as it does not require wearing masks and allows for a fuller use of nonverbal communication.

##### 3.2.1.3 Flexibility

The third group of advantages of online therapy referred to the flexibility of this form of work and was indicated as an advantage by a total of 11.65% respondents. The most frequently mentioned subcategory was flexibility in the schedule (9.89%), referring to the ability to adjust the time of sessions to individual needs. The possibility of adapting to crisis situations (1.41%) and conducting sessions at unusual times (0.35%) were indicated much less frequently. Therapists emphasized that the remote form makes it easier to adjust the dates of sessions to the changing needs of patients, which helps to maintain the continuity of therapy and reduces the number of canceled appointments.

##### 3.2.1.4 Cost reduction and practicality

The fourth group of advantages is cost reduction and practicality, which was indicated by a total of 6% respondents. The most frequently mentioned subcategory was cost efficiency, referring to the elimination of travel costs, which was indicated by 3.53% people. The second subcategory was the minimization of organizational requirements, i.e. no need to rent a practice and simplification of work logistics, which was indicated by 2.47% people. Therapists have repeatedly emphasized that online therapy allows for cost savings, both on the part of the patient and the specialist. Online psychotherapy has also been appreciated due to the simplification of the logistics of therapeutic work, which is manifested, among others, in the lack of the need to rent an office and the freedom to choose the place of work.

##### 3.2.1.5 Comfort and familiarity with the environment

The fifth group of advantages of online psychotherapy concerned comfort and knowledge of the environment. This advantage was indicated by 12% of respondents. Attention was paid to both the comfort (8.12%) and the sense of security (3.88%) associated with the patient’s sessions from home. It should be noted that comfort is more about external and practical conditions (comfort, familiar space), while the sense of security refers to the emotional state of the patient, conducive to trust, openness and reduction of tension. Both elements complement each other and affect the quality of the therapeutic relationship in the context of online sessions.

##### 3.2.1.6 Functionality in online diagnosis and therapy

The sixth group of advantages of online therapy is functionality in online diagnosis and therapy (10.94%). A qualitative analysis of the psychotherapists’ statements indicates that the study participants saw clear benefits from functional aspects that support both the diagnosis and the organization of therapeutic work. Psychotherapists pointed out that the ability to observe the patient in his or her natural environment provides a valuable context for understanding his or her functioning. They pointed out that changing the context of the session to a home one brings new threads to the conversation and allows for a more holistic view of the patient.

At the same time, it was emphasized that the online form is conducive to better planning and monitoring the structure of the session. Psychotherapists noticed that online meetings are easier to plan, more punctual and orderly, which increases the effectiveness of therapeutic work.

Another important element supporting online therapy was also the possibility of convenient sharing of psychoeducational materials. The transfer of information in this form is quick and technically simple, which allows for the effective inclusion of psychological education in the therapeutic process.

All these statements show that the remote form of therapy not only maintains the quality of the therapeutic relationship, but also enriches it with new, practical possibilities – conducive to diagnosis, planning and integration of educational content.

#### 3.2.2 Disadvantages of online therapy noticed by psychotherapists

Online therapy, although it has many advantages, is also associated with a number of limitations and disadvantages noticed by psychotherapists. The areas they indicated were: technical problems, problems with the effectiveness of this form of therapy, organizational difficulties, and problems related to confidentiality and security (**Table 3**). It is worth noting that some of these difficulties were perceived as common to both sides of the therapeutic process, others as particularly troublesome for patients, and still others as specific challenges only for therapists.

**Table 3:** Disadvantages of online therapy noticed by psychotherapists.

Main category	Subcategory	Description	N	%
1. Efficiency Issues	1.1. Weakening of the therapeutic relationship due to lack of physical contact	Weakening of the therapeutic relationship due to lack of physical contact	70	24.73%
	1.2. Difficulty in diagnosing and recognizing the problem	Difficulty diagnosing and recognizing the problem	34	12.01%
	1.3. Communication problems	Communication problems	27	9.54%
	1.4. Limitation of the possibility of using certain therapeutic techniques	Limitation of the possibility of using certain therapeutic techniques	26	9.18%
	1.5. Problems with the therapist’s concentration	Problems with the therapist’s concentration	11	3.88%
	1.6. Convincing the therapist of lower effectiveness (or less effectiveness)	Convincing the therapist of less effectiveness (or less effectiveness)	11	3.88%
	1.7. Limited access to emergency assistance	Limited access to emergency care	8	2.82%
	1.8. Less sense of agency in therapists	Less sense of agency in therapists	4	1.41%
2. Technical Issues	2.1. Lack of stable connection	No stable connection	53	18.72%
	2.2. Hardware or software failure	Hardware or software failure	4	1.41%
	2.3. Incomprehensible sound	Incomprehensible sound	4	1.41%
3. Security Issues	3.1. Fear of breach of session secrecy	Fear of violating session secrecy	34	12.01%
	3.2. Fear of the presence of third parties	Concern about the presence of third parties	18	6.36%
	3.3. Concern about recording the session	Concern about recording sessions	2	0.70%
4. Organizational difficulties	4.1. Problems with concentration of patients	Problems with concentration of patients	9	3.18%

##### 3.2.2.1 Efficiency issues

The first group of disadvantages of online therapy noticed by psychotherapists relate to overall therapy effectiveness. As many as 67.45% of respondents indicated this category, which proves a clear belief in the limitations of the effectiveness of this form of work. Among the most frequently mentioned problems were: weakening of the therapeutic relationship due to the lack of physical contact, difficulties in diagnosing and recognizing problems, problems with communication, limitations in responding to crisis situations, problems with concentration on the part of the therapist, belief in the lower effectiveness of this form of therapy, limitation of the possibility of using certain therapeutic techniques and reduced sense of agency. All these aspects can affect the quality and course of the therapeutic process to varying degrees.

One of the most frequently indicated aspects related to effectiveness was the weakening of the therapeutic relationship resulting from the lack of physical contact, which was indicated by 24.73% of respondents. Qualitative analysis of the results shows that many therapists emphasized that being present in one physical space is conducive to building bonds and trust, which are the foundation of effective therapy. The lack of the possibility of a face-to-face meeting may make it difficult to establish a deep therapeutic relationship, especially at the initial stages of working with a new patient. Another significant disadvantage of online therapy turned out to be the difficulty in properly identifying the patient’s problem and making an accurate diagnosis via the Internet (12.01%). Lack of direct contact and limitations in observing non-verbal behavior may lead to misinterpretations or difficulties in fully understanding the patient’s situation. According to psychotherapists, the diagnostic process, which is a significant stage of therapeutic work, often requires careful observation, including various subtle signals, which may be unavailable to a psychotherapist in a remote context Pointing to the disadvantages of online therapy, psychotherapists also noted communication challenges (9.54%). Psychotherapists paid special attention to the impoverishment of the message in terms of the so-called metamessages, i.e. elements of non-verbal and paraverbal utterances (e.g. tone of voice, facial expressions, gestures, pauses, pace of speech), which give additional meaning and context to the verbal content of the utterance. Psychotherapists pointed out that a direct meeting with the patient allows for intuitive sensing of changes in facial expressions, body tension or microgestures, which are often a signal of deeper experiences or internal conflict. In the conditions of remote therapy, non-verbal signals are much more difficult to notice. Therapists were afraid that if the message is filtered through technology, there may not only be problems with the therapist’s misunderstanding of the patient, but that the perception of them as psychotherapists will also be disrupted. The analysis of statements also results in the fear of losing the natural flow of the conversation. Therapists pointed to the difficulty in creating a dialogue, which in the classic format is often based on a delicate consonance of speech, changing the roles of the speaker and the listener, as well as joint emotional regulation. These difficulties, according to psychotherapists, translated directly into a reduction in the naturalness and spontaneity of contact, which, in their opinion, may affect the sense of artificiality and distance.

Some therapists (9.18%) pointed to limitations on using certain techniques. This mainly concerned methods that required work with the body, symbols or physical space. Therapists were also concerned about the use of more confrontational interventions in remote therapy, and also pointed out the inability to use supporting materials such as flipcharts, whiteboards or worksheets.

On the other hand, slightly less frequently mentioned disadvantages of online therapy, related to its effectiveness, were, among others, difficulties with maintaining concentration by psychotherapists during online sessions, which was mentioned by 3.88% of respondents. Some therapists indicated that they are more easily distracted when working remotely, which may translate into the quality of their sessions and their effectiveness. Some were also convinced that online therapy was less effective (3.88%). In their statements, they expressed doubts as to whether remote work allows to achieve the same results as face-to-face meetings. On the other hand, about 2.82% of the respondents indicated a limited ability to provide emergency assistance. A qualitative analysis of their statements shows that in situations requiring quick therapeutic intervention, the lack of a specialist’s physical presence and the limitations resulting from the nature of online contact may make it difficult to take appropriate action.

The last of the problems mentioned related to the effectiveness of online psychotherapy was the general sense of agency in therapists during this form of work. This issue was signaled by 1.41% of the psychotherapists surveyed.

##### 3.2.2.2 Technical issues

The second group of disadvantages of online psychotherapy mentioned by psychotherapists was the technical problem category. It constituted a significant limitation in conducting online therapy, affecting both the comfort of conducting sessions and the effectiveness of psychotherapy. Among the technical problems, the therapists noticed the problem with: lack of a stable internet connection, hardware or software failure, as well as poor sound quality. Which is troublesome for both the patient and the psychotherapist.

The most frequently reported technical problem was unstable internet connection (18.72%). It was emphasized that an unstable connection can lead to session interruptions, delays in communication and difficulties in the flow of conversation. These problems were particularly noticeable in places with limited network infrastructure. Although the most frequently mentioned disadvantage of online therapy, as part of technical problems, was the instability of the Internet connection, some therapists also indicated concern about hardware and software failures and difficulties related to the sound quality during the conversation (1.41%). In their opinion, such difficulties may lead to the need to interrupt or postpone the session, which negatively affects the continuity of the therapeutic process. Equipment failure in moments of greater emotional tension can disrupt the course of therapy, making it difficult to provide emotional support to the patient.

An additional problem was poor sound quality, which was also pointed out by 1.41%. It can significantly hinder mutual understanding of the participants of the session, and in extreme cases completely prevent the conversation. It was pointed out that although it does not experience significant technical problems, there are occasional disruptions that can affect the flow of communication.

In conclusion, hardware failures, software failures and audio problems pose significant challenges for online therapy. These disruptions can negatively affect the quality of interaction, session confidentiality and communication flow, which consequently reduces the effectiveness of therapeutic intervention. These results indicate the need to implement technological and emergency solutions that will minimize the risk of disruption and ensure greater stability of the therapeutic process.

##### 3.2.2.3 Security issues

The third group of disadvantages of online psychotherapy is the category related to safety, which was mentioned by 19.07% of respondents. The most frequently indicated defect in this category was the fear of violating the secrecy of the session, reported by 12.01%. Psychotherapists expressed clear concerns about the possibility of violating the secrecy of sessions in online therapy settings, noting the lack of control over the patient’s environment and technical threats to confidentiality, such as the possibility of interception of transmissions. Another disadvantage, related to safety, was the concern about the presence of third parties in the space where the patient participates in the session (e.g. household members, roommates) (6.36%). Their statements show that patients – especially children and adolescents – often do not have a safe space at home to talk, and the presence of parents, or, in the case of adults, their partners or children, can disturb the course of the meeting. In addition, there was a concern (0.70%) about the possibility of recording sessions without the therapist’s knowledge and consent, which, despite its low frequency, poses a significant threat to the privacy and ethical standards of psychotherapeutic work.

##### 3.2.2.4 Organizational problems

The last group of disadvantages of online therapy were concerns about organizational issues, which included problems with the patient’s concentration on the online session (3.18%).

Statements by psychotherapists indicate that patients find it difficult to focus during online sessions, especially in the case of children. Therapists noted that patients were easily distracted due to the presence of household members, space limitations, as well as the impact of electronic devices, which leads to a decrease in the quality of contact and the loss of important information.

The above-mentioned limitations and disadvantages of online therapy relate to both the effectiveness and safety, as well as the organization of sessions conducted remotely. The information obtained is a valuable field for discussion and consideration as to the scope of work on eliminating difficulties that arise in online therapy, as it clearly indicates the direction, which should result in the development of specific guidelines, taking into account the reported concerns and dilemmas.

### 3.3 Quantitative data

In accordance with the adopted research model, in order to ensure the clarity and transparency of the presented results, the description of the quantitative data analysis is presented as follows: first, (a) the assessment of the advantages of online therapy in the context of the decision to use this form of work, then (b) the relationship between the perception of the advantages of online therapy and the decision to use it, taking into account potential emotional and epidemiological-institutional mediating factors. The next point presents in a similar way (c) the assessment of the disadvantages of online therapy in the context of the decision to use it and (d) in the context of potential mediators.

#### 3.3.1 Seeing the advantages of online therapy and the decision to use this form of work

In order to examine the relationship between the perception of the advantages of online therapy and the current conduct of online therapy (**Table 4**), a chi-square independence test was performed. The analysis showed no statistically significant relationship between these variables, χ²(1) = 1.24, p = .266. The Phi coefficient was.07, which indicates a very weak strength of the relationship. These results suggest that the perception of the advantages of online therapy is not significantly related to the current conduct of therapy by therapists.

**Table 4:** Seeing the advantages of online psychotherapy by psychotherapists and the current conduct of online therapy.

Seeing the benefits of online therapy	Current online therapy		N (%)
	Yes	No	
Yes	226	33	259 (91.5%)
NO	19	5	24 (8.5%)
N (%)	245 (86.6%)	38 (13.4%)	283 (100%)

Despite the lack of a significant relationship detected by the χ² test, it was decided to use regression analysis because the χ² test only examines the general relationship between categorical variables, providing no information about the direction, strength of the relationship, or possible intermediate variables. Hence, in the next step, it was decided to check whether the perception of the advantages of online therapy influences the decision to use it, by conducting a logistic regression analysis, in which the dependent variable is the conduct of online therapy (0 = no, 1 = yes), and the predictor is the perception of the advantages of online therapy (0 = NO, 1 = YES). The regression model did not reach the level of statistical significance (β = 0.91, SE = 0.62, Wald z = 1.45, p = .146). The odds ratio was OR = 2.48, 95% CI [0.73, 8.46]. Although this result seems to indicate a tendency suggesting that therapists who perceived advantages might be more likely to provide online therapy, the effect did not reach statistical significance, and the wide confidence interval reflects substantial uncertainty of the estimate (**Table 5**). It should be emphasized, however, that because the study sample is not representative and is not equal in terms of the number of individuals using and not using remote therapy, the interpretation of the obtained results should be approached with caution.

**Table 5:** The impact of perceiving the advantages of online therapy on the decision to use it.

Variable	B	HERSELF	Forest z	p	OR	95% CI OR
Advantages of online therapy (1 = YES, 0 = NO)	0.907	0.624	1.454	.146	2.478	[0.726, 8.458]

β, logistic regression coefficient; SE, standard error; OR, odds quotient; CI, 95% confidence interval for OR.

In order to refine the analyses and investigate whether the specific advantages of online therapy can predict the decision to use it, 20 separate logistic regression analyses were conducted, in which the decision to conduct online therapy was the dependent variable (0 = no, 1 = yes) and the predictor was the presence of a given advantage (1 = indicated, 0 = no) (**Table 6**).

**Table 6:** Influence of individual advantages of online therapy on the decision to use it.

No	Advantages	B	OR	p
1	Z_1.1. Geographical flexibility	–0.116	0.89	.745
2	Z_1.2. Continuity of Care	0.130	1.14	.749
3	Z_1.3. Time saving	0.992	2.70	.112
4	Z_2.1. Protection against infection	0.192	1.21	.707
5	Z_3.1. Flexibility in Schedule	–0.108	0.90	.818
6	Z_3.2. Adapting to Crisis Situations	–0.342	0.71	.607
7	Z_3.3. Possibility of Sessions at Unusual Times	0.525	1.69	.425
8	Z_4.1. Cost Efficiency	0.094	1.10	.910
9	Z_4.2. Minimum organizational requirements	0.052	1.05	.957
10	Z_5.1. Patient Comfort at Home	–0.287	0.75	.579
11	Z_5.2. A sense of security at home	–0.162	0.85	.742
12	Z_6.1. Insight into the Patient’s Environment	0.378	1.46	.576
13	Z_6.2. Session structure	–0.636	0.53	.439
14	Z_6.3. Possibility of providing psychological education	–0.201	0.82	.726

All univariate models (Y, decision to treat online; X, indication of a given advantage); β, logistic regression coefficient; OR, odds ratio; p, significance value.

Ultimately, none of the studied advantages showed a statistically significant relationship with the decision to conduct online therapy (all p >.05). The strongest, though insignificant, trend was observed for the advantages of (Z_1.3) “time saving” (β = 0.99, OR = 2.70, p = .112), which may suggest that therapists valuing this aspect were more likely to provide online therapy. However, this hypothesis should be treated with caution because the effect did not reach the level of statistical significance (**Table 5**).

##### 3.3.1.1 Emotional and epidemiological-institutional factors as potential mediators in the relationship between the perception of the advantages of online therapy and the decision to use it

Despite the lack of a direct effect (37), guided by the principle that the mediating effect can manifest itself even when the total effect is not statistically significant (38), the next step was to check whether selected emotional and epidemiological-institutional variables mediate the relationship between the perception of the advantages of online therapy and the decision to use it, A mediation analysis using the bootstrap method was performed (2–000 replicas, 95% confidence intervals). The predictor variable was the dichotomous variable “advantages” (0 = no, 1 = yes), the dependent variable was the decision to conduct online therapy (0 = no, 1 = yes).

The following were taken into account as emotional mediating variables: the level of fear of COVID-19, anxiety symptoms (HADS-A) and depressive symptoms (HADS-D), pandemic fatigue in two dimensions – informational and behavioral. In addition, five main epidemiological and institutional variables were analyzed, which the respondents could indicate as the reasons for the decision on the form of therapy: the epidemiological situation, government recommendations, recommendations of psychotherapeutic societies, the patient’s health condition and the therapist’s health status (**Table 7**).

**Table 7:** Indirect effects for emotional and epidemiological-institutional mediators in the relationship between the perception of the advantages of online therapy and the decision to use it.

No	Mediator	Indirect effect (a·b)	95% CI under	95% CI upper	p
1	Fear of COVID-19	0.020	–0.154	0.212	No
2	Symptoms of Anxiety (HADS-A)	0.029	–0.257	0.260	No
3	Symptoms of Depression (HADS-D)	–0.014	–0.219	0.213	No
4	Information fatigue (pandemic)	–0.027	–0.208	0.169	No
5	Behavioral fatigue (pandemic)	–0.003	–0.223	0.205	No
6	Epidemiological situation (code 1)	–0.030	–0.143	0.073	No
7	Government recommendations (code 2)	–0.026	–0.132	0.082	No
8	Recommendations of psychotherapeutic societies (code 3)	–0.061	–0.210	0.042	No
9	Patient’s health status (code 4)	0.029	–0.084	0.155	No
10	Therapist’s health status (code 5)	0.010	–0.055	0.091	No

The results showed that none of the mediators analyzed had a statistically significant mediating role. All 95% confidence intervals for the product of the a·b pathways (indirect effect) contained zero, indicating no mediation. The highest score values were recorded for emotional variables – m.in. for anxiety about COVID-19 (a·b = 0.020, 95% CI [–0.154, 0.212]) and general anxiety (a·b = 0.029, 95% CI [–0.257, 0.260]) – but these effects also did not reach significance. Epidemiological-institutional variables, such as government recommendations (a·b = –0.026, 95% CI [–0.132, 0.082]) or the epidemiological situation (a·b = –0.030, 95% CI [–0.143, 0.073]), also did not show mediating effects (**Table 6**).

These results suggest that the perception of the benefits of online therapy works independently of the therapist’s psychological variables or institutional reasons indicated as motives for the decision to use online therapy. It can therefore be assumed that a positive perception of online therapy directly motivates its use, but does not act through mediating variables such as anxiety, fatigue or external guidelines.

#### 3.3.2 Noticing the disadvantages of online therapy and the decision to use this form of work

Similarly, the first analysis of the relationship between the perception of disadvantages in online therapy (**Table 8**) and the current conduct of online therapy using the chi-square independence test was carried out. The results of the analysis did not show a statistically significant relationship between these variables, χ²(1) = 1.04, p = .309. The strength of the dependency was very weak (Phi = .06). This means that the fact that therapists notice the disadvantages of online therapy is not significantly related to whether they are currently conducting online therapy.

**Table 8:** Noticing the disadvantages of online psychotherapy by psychotherapists and the current conduct of online therapy.

Spotting the flaws of online therapy	Current online therapy		N (%)
	Yes	No	
Yes	211	35	246 (86.9%)
NO	34	3	37 (13.1%)
N (%)	245 (86.6%)	38 (13.4%)	283 (100%)

The next step was to assess whether noticing the disadvantages of online therapy affects the decision to use it. Similarly, a logistic regression analysis was performed. The model was also found to be statistically insignificant (β = –0.63, SE = 0.63, Wald z = -1.00, OR = 0.53, p = .316) (**Table 9**).

**Table 9:** Impact of the perception of the disadvantages of online therapy on the decision to use it.

Variable	B	HERSELF	Forest z	p	OR	95% CI OR
Disadvantages of Online Therapy (1 = YES, 0 = NO)	–0.631	0.629	–1.00	.316	0.532	[0.155, 1.826]

β, logistic regression coefficient; SE, standard error; OR, odds quotient; CI, 95% confidence interval for OR.

Despite the lack of significance of the relationships, a series of logistic regressions with a dichotomous dependent variable (1 = currently leading online therapy, 0 = not leading) were again performed to analyze in detail whether specific perceived disadvantages of online therapy affect the decision to use it. In this case, thirteen of the most frequently indicated defects of online therapy were used as predictors, of which only those reported by at least 10 respondents were qualified for further analysis (to ensure the stability of the estimate). Finally, seven categories were taken into account: difficulty in diagnosing and recognizing the problem, weakening of the therapeutic relationship, communication problems, limitation in the possibility of using certain therapeutic techniques, problems with concentration, fear of violating the secrecy of the session and the presence of third parties during the session.

The results showed that none of the analyzed defects was a statistically significant predictor of the decision to conduct online therapy (all p >.19). Although some predictors (e.g., difficulty with diagnosis; b = –0.60, OR = 0.55, p = .197) tended to have an effect, this effect did not reach the level of significance. The results suggest that individual aspects of perceived difficulties did not clearly determine the decision on the choice of the form of therapy, and other, more complex or external, factors may have had an impact (**Table 10**).

**Table 10:** Impact of perceived disadvantages of online therapy on the decision to use it.

No	Disadvantages	B	OR	p
1	W_1.1 Weakening of the therapeutic relationship due to lack of physical contact	0.239	1.27	.573
2	W_1.2. Difficulty diagnosing and recognizing the problem	–0.601	0.55	.197
3	W_1.3. Communication problems	0.237	1.27	.711
4	W_1.4 Limitation of the use of certain therapeutic techniques	–0.176	0.84	.759
5	W_1.5. Trouble concentrating	0.454	1.57	.670
6	W_3.1. Fear of violating session secrecy	0.256	1.29	.618
7	W_3.2.Concern of the presence of third parties	–0.081	0.92	.898

All single-factor models; β, logistic regression coefficient; OR, odds ratio; p, significance value.

##### 3.3.2.1 Psychological and epidemiological-institutional factors as potential mediators in the relationship between the perception of the disadvantages of online therapy and the decision to use it

Again, despite the lack of a direct effect (37), an analysis of potential indirect effects was performed, taking into account the distinguished psychological and epidemiological-institutional potential mediators. For each of these variables, an intermediate effect (a·b) was calculated along with a 95% confidence interval based on bootstrapping (500 replicas), where the predictor variable in this case was the dichotomous variable for perceiving the flaws of online therapy (0 = no, 1 = yes), the dependent variable, again the decision to conduct online therapy (0 = no, 1 = yes).

In none of the cases, taking into account psychological variables, the range excluded zero, indicating the absence of significant mediating effects. This means that the above factors did not explain the relationship between the perception of the disadvantages of online therapy and the decision to use it (**Table 11**).

**Table 11:** Indirect effects on psychological and epidemiological-institutional mediators for the relationship between the perception of the disadvantages of online therapy and the decision to use it.

No	Mediator	Indirect effect (a·b)	95% CI under	95% CI upper	p
1	Fear of COVID-19	0.188	–0.169	0.645	No
2	HADS-A (symptoms of anxiety)	–0.064	–0.744	0.499	No
3	HADS-D (symptoms of depression)	0.021	–0.242	0.266	No
4	Information fatigue	0.128	–0.182	0.460	No
5	Behavioral fatigue	–0.003	–0.412	0.347	No
6	Epidemiological situation	–0.011	–0.104	0.073	No
7	Government recommendations	0.114	0.001	0.296	**Yes**
8	Recommendations of psychotherapeutic societies	–0.184	–0.414	–0.002	**Yes**
9	Patient’s health status	0.081	–0.016	0.252	No
10	Therapist’s health condition	–0.002	–0.078	0.065	No

**Figure 1:**
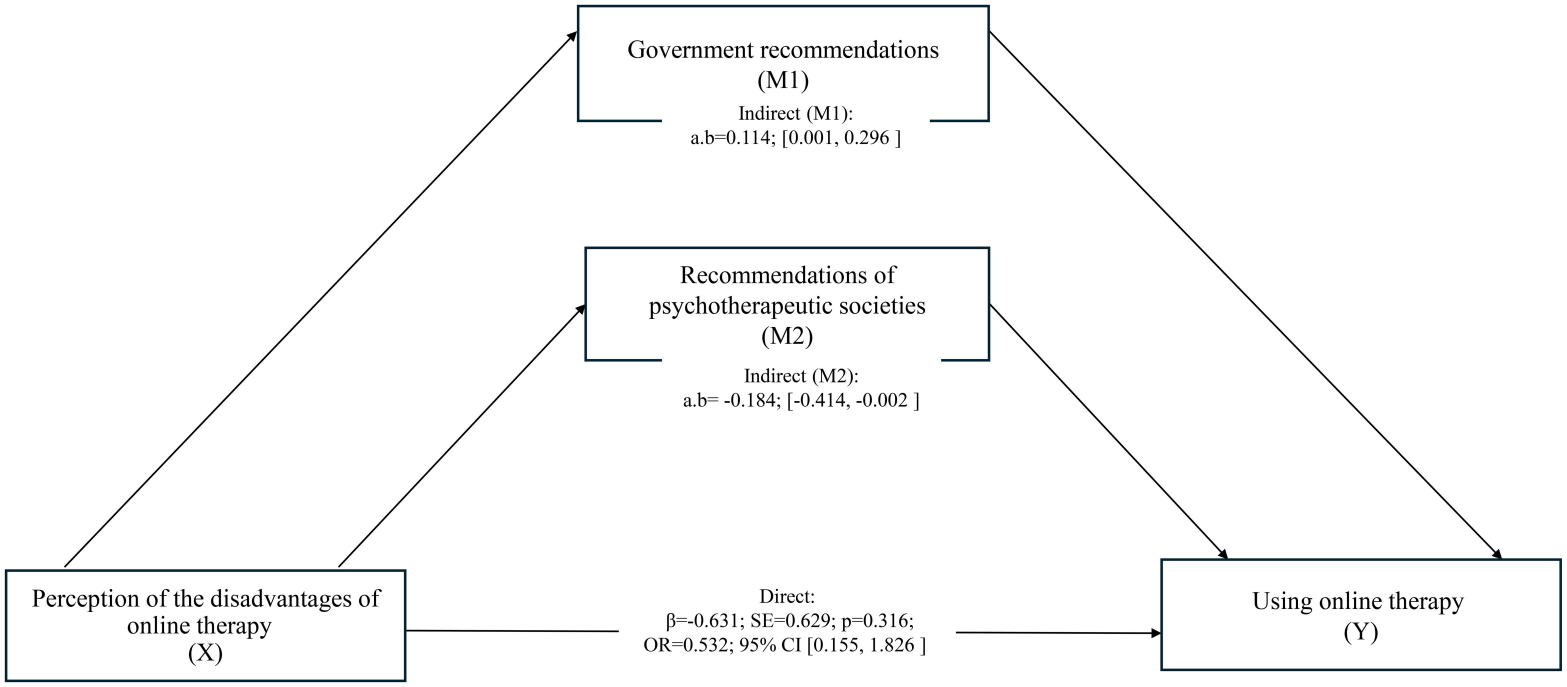
Mediation model for the disadvantages → decision pathway. Significant indirect effects: government recommendations (positive), psychotherapeutic society recommendations (negative). Emotional mediators were non-significant.

On the other hand, among the analyzed epidemiological-institutional intermediate variables, two factors showed statistically significant indirect effects. Indicating government recommendations as the reason for the decision was associated with a significant positive indirect effect: a·b = 0.114, 95% CI [0.001, 0.296]. On the other hand, recommendations of psychotherapeutic societies showed a significant negative effect: a·b = –0.184, 95% CI [–0.414, –0.002] (**Table 11**). The results of the analysis are shown in **Figure 1**.

In mediation models linking cognitive appraisals to the decision to conduct online psychotherapy, no indirect effects emerged for the “advantages → decision” pathway across emotional (COVID-19 fear, HADS-A, HADS-D, pandemic fatigue) or epidemiological–institutional mediators (all 95% CIs included zero). For the “disadvantages → decision” pathway, two institutional mediators showed significant indirect effects in opposite directions: (i) government recommendations yielded a small positive indirect effect (a·b = 0.114, 95% CI [0.001, 0.296]), increasing the probability of adopting online therapy despite perceived drawbacks; (ii) psychotherapeutic society recommendations yielded a small negative indirect effect (a·b = −0.184, 95% CI [−0.414, −0.002]), decreasing the probability of adoption when drawbacks were salient. Emotional mediators were non-significant throughout.

Institutional factors, although seemingly similar - two types of recommendations - can have a psychologically different effect. Government recommendations increased the conformity of behavior with social expectations, despite internal doubts, while environmental recommendations seemed to reinforce individual skepticism in the context of professional standards.

## 4 Discussion

Most therapists reported both benefits and drawbacks of online therapy.

Although online therapy first surged as a pandemic response, it rapidly became a genuine means to expand access to care. Analyzing its advantages, the therapists paid attention to: the ease of participation in sessions from anywhere, the possibility of continuing treatment, despite barriers such as distance or isolation, or time flexibility. However, remote sessions are associated with significant challenges, such as: unstable Internet connections or technical problems that may interrupt therapy, limited access of the psychotherapist to non-verbal signals, which may weaken communication and, consequently, prevent the possibility of building a deep therapeutic bond. A detailed analysis of these limitations reveals which aspects of remote therapy underperform and suggests areas for improvement. Finally, analyzing these elements gives you a chance to formulate practical recommendations: setting minimum technical standards (hardware, software, bandwidth), introducing routine connection tests before the session, or recommending specialized training for therapists in effective online work.

### 4.1 Advantages of online therapy

**Figure 2:**
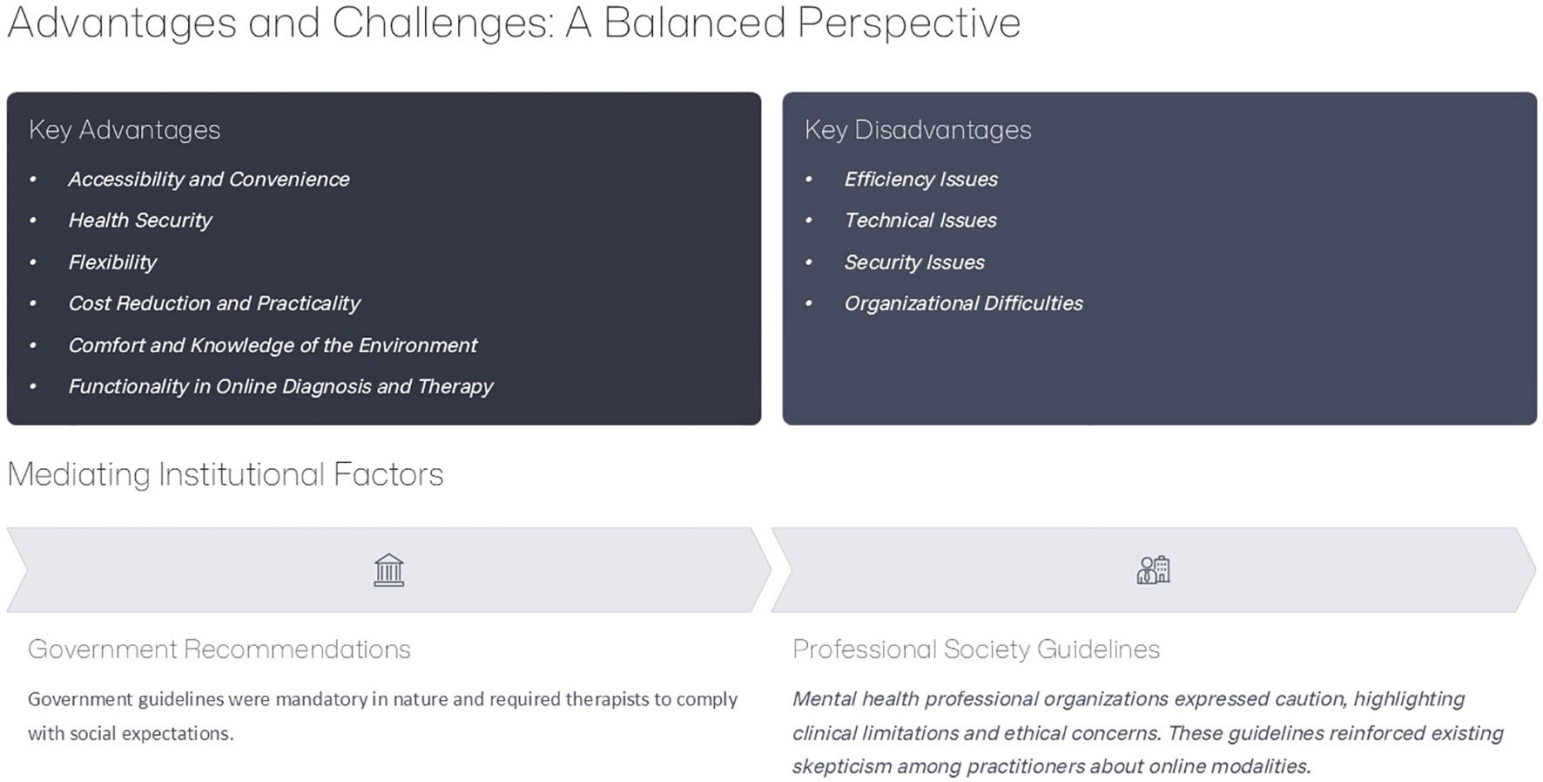
Online Therapy: Advantages, Disadvantages and Mediating Effect.

The conducted research shows that an important advantage of online psychotherapy is its greater availability and flexibility, which translates into time savings, cost reduction, but also geographical and organizational flexibility (see **Figure 2**). These factors, from the point of view of the lockdown and the restrictions on social contacts imposed during the COVID-19 pandemic, seem to be of great importance for maintaining the smoothness of the therapeutic process and greater access to therapy (17, 39, 40). And this, from the patient’s perspective, can also be a factor in reducing the level of stigma (41, 42). From a therapist’s perspective, providing more flexibility in the workplace can act as a protective factor in reducing burnout (43).

Psychotherapists also drew attention to the aspect of health safety and reducing the risk of infection thanks to online sessions. In the era of the pandemic, health protection and prevention of the spread of the virus were one of the fundamental aspects that everyone was obliged to pay attention to on a regular basis. The risk of infection, higher levels of fear of COVID-19 infection and health complications were associated with anxiety about oneself and one’s loved ones (44, 45), as well as appreciating the greater benefits of online therapy anonymity (46). In the health context, it seems that the aspect of health security and reducing the risk of infection has a broader context, going beyond the pandemic period. After all, similar concerns can accompany therapy on a daily basis, working with patients with symptoms of various infectious diseases or struggling with reduced immunity. Online therapy is therefore a real answer to the need for physical distancing.

Functionality in diagnosis and therapy conducted remotely was also emphasized. The online form is to foster better planning of the session structure, and also gives the opportunity to use digital psychoeducational materials in a quick and technically simple way. Without a doubt, this expands the possibilities of work. Recent research (47) on mental health based on AI also highlights the transformative potential of technology in the delivery of mental health care, suggesting that artificial intelligence (AI) provides innovative solutions geared toward enhancing certain psychotherapeutic interventions. Nevertheless, further extensive research is needed on the practical and ethical aspects of the use of AI in mental health care.

The online form of work also gives the opportunity to observe the patient and his reactions in his natural environment, which is a new source of information to which therapists have not had any access so far. These new threads can complement a holistic view of the patient. In addition, some studies suggest that patients themselves rate that they feel more comfortable during seya while being in their own homes (48, 49).

### 4.2 Disadvantages of online therapy

Nevertheless, although the surveyed psychotherapists pointed to a number of benefits of online sessions, there is no doubt that it is not free from disadvantages and limitations (see **Figure 2**). The most frequently raised objection concerned broadly understood effectiveness, as more than half of the respondents paid attention to it. Interestingly, these doubts are contrary to current scientific research (12, 50, 51), which mostly shows that both face-to-face therapy and online therapy are equally effective. However, some studies have shown that some theoretical orientations may be better suited to the online work form than others (12, 52–54). Perhaps the lack of previous experience and self-assessment may also raise concerns about its effectiveness, as research shows that therapists with experience in online interventions even before the COVID-19 pandemic, had more positive attitudes toward online psychological interventions (17, 55). The psychotherapists surveyed also have concerns about the weakening of the therapeutic relationship. Current scientific research suggests that since the COVID-19 pandemic, there has been an increased appreciation for online therapy and the possibility of building a fruitful therapeutic relationship in this form of work (51, 56, 57). Nevertheless, other analyses indicate that relationships in online therapy are generally weaker than in face-to-face therapy (58) and that the relationship between therapist and patient is rated as more effective and productive in face-to-face therapy in terms of positive cooperation as well as positive clinical contribution (59). And yet the therapeutic relationship is one of the most important factors in the treatment process (60). Perhaps, when analyzing inconsistent scientific reports and in the absence of personal previous experience with online therapy, psychotherapists are particularly cautious about excessive optimism and positive assessment of it. Further research is needed to explain in detail the level of effectiveness of remote therapy and the effectiveness of its specific elements, particularly important for the process, such as the therapeutic relationship, compared to face-to-face therapy, taking into account the specificity of individual theoretical modalities. At the same time, psychotherapists should be encouraged to constantly monitor their own attitude toward online therapy, their own emotions related to it, dilemmas, fears or challenges, and how these factors may affect the assessment of online therapy.

Another reported aspect was the limitations as to the ability to respond adequately in crisis situations or difficulties with diagnosing and recognizing the problem, which may be related to the inability to monitor the client globally, including non-verbal communication (12, 61, 62). Affect, eye contact, and psychomotor functioning are important components of mental state testing (56). The therapist should notice their client’s emotions and respond to them appropriately, hence there may be concerns about overlooking important diagnostic information if sessions are held remotely (63). Lack of control in relation to dealing with risk-related situations also increases therapists’ anxiety (64). It seems that it may be important to have an explicit contract for online counseling (65), which is intended to serve lower stress levels but also to increase the therapeutic process as a whole (17). Establishing a specific procedure in a crisis situation at the level of the contract with the patient can give a sense of a specific framework of possible interventions and a strategy for the actions taken.

Attention was also drawn to the lack of possibility of using the entire spectrum of therapeutic techniques. Thus, the previously well-known structure requires therapists to flexibly adapt to the virtual environment. In the era of the pandemic, when psychotherapeutic societies opened up to online work (cf. 45), therapists faced the challenge of moving their work into a new space. Flexibility and adaptability in the use of therapeutic interventions require reflection on the quality of therapy, as well as appropriate adaptation of the process to the effective use of online tools and techniques (66). It seems that training conducted by psychotherapeutic institutions and associations in the field of digital competence development, focusing on the possibilities of conducting online sessions with a focus on adapting therapeutic techniques of individual psychotherapeutic modalities to the virtual space, could be helpful.

Problems with concentration and a lower sense of agency on the part of the therapist are other disadvantages of online therapy reported by them. Research confirms that therapists are more prone to distractions in settings other than face-to-face (67, 68). Interestingly, with a decrease in the reported challenges of online therapy over time, which is argued m.in the adaptation process, an increase in the level of distraction is observed (cf. 68). Also, the therapist’s experience in online counseling and their comfort during the session (68) will be important for the sense of security (69). Békés and Aafjes-van Doorn (17) pointed out that although therapists from different parts of the world have been successful in finding their way in online therapy, both therapist fatigue and lack of self-confidence are factors influencing attitudes toward telepsychotherapy. The growing experience of psychotherapists in remote work, the exchange of experiences between specialists, as well as supervision, support and training in remote sessions, and the development of effective forms and working conditions that will strengthen the sense of control and agency of therapists, may be helpful in reducing the above difficulties and caring for the psychological comfort of the therapists themselves.

Another group is technical problems that can hinder the quality and stability of the session. The area of technical problems or lack of knowledge of the tools used for remote work has already been reported in previous scientific reports (40, 65), which suggests how important this point is when talking about remote therapy and how important education and training in new technologies and safety in online therapeutic work are becoming. Technology Acceptance Model (TAM) (70, 71) explains that the use of technology depends not only on the assessment of its effectiveness, but also on the subjective assessment of the ease with which it can be used. Knowledge and previous experience give you the opportunity to properly use the possibilities of the Internet in the future (72). So far, as part of psychotherapist training, no space has been devoted at all to aspects of remote work possibilities. Online training courses, although considered necessary and beneficial, are not a permanent training offer (73, 74). It is not surprising that there is a lack of knowledge and guidelines that could be a signpost for the work of therapists in the virtual space. The emerging recommendations and guidelines are still being modified, hence they may cause concern. It would be important to implement comprehensive training in technology in psychotherapeutic work and specific guidelines that provide real reference points for the possibility of remote work, taking into account specific therapeutic modalities and the work tools used in them. Defining specific frameworks and comprehensive work protocols will define online counseling (75), giving therapists a reference point and specific criteria for possible action.

Security issues are associated with concerns about limiting confidentiality. And here, on the one hand, it can be pointed out that not everyone has the opportunity to create such a space at home that will ensure confidentiality during remote sessions (40, 76–78). Therapists also pay attention to a number of distractions that can affect the level of concentration of their patients, especially in the case of sessions conducted with children. On the other hand, there are reports of digital security breaches by large tech companies in the public space (cf. 79, 80), which may also raise concerns about the level of data security. It is worth noting, however, that the reported concerns are consistent with analyses carried out before the pandemic (cf. 12, 81), which may suggest that confidentiality and privacy are among the fundamental areas about which there are still concerns as part of remote therapy. Furthermore, recent global analyses also emphasize that beyond clinical effectiveness, telepsychology raises significant legal and ethical issues that must be addressed in mental health practice (82)Besides, the lack of a sense of security, privacy and confidentiality can significantly affect the level of disclosure of personal content and, consequently, affect the effectiveness of therapy (80). To sum up, it seems important to implement specific guidelines and good practices that will focus on minimizing technological risk. Which may include therapeutic contact, educating patients on how to prepare the appropriate environment for a remote therapeutic session. A focus on the security and confidentiality of the data and the location of the therapy session can minimize the concerns raised.

### 4.3 Determinants of the decision to use online therapy

Research has shown that the perception of the advantages and disadvantages of online therapy by psychotherapists is not directly related to the decisions they make about its use. However, it turns out that two factors mediate the relations between noticing the disadvantages of online therapy and using this form of work: government recommendations and recommendations of psychotherapeutic associations (see **Figure 2**).

The government recommendations acted as a factor facilitating the decision to use online therapy, despite the therapist’s negative beliefs about this form of work. Therapists who saw online therapy as flawed but cited government recommendations as the reason for their decision were more likely to use it. This may result from the need to justify the choice by appealing to an external authority. Government recommendations could have a normative function and serve to reduce cognitive dissonance, enabling the therapist to reconcile internal doubts with the need to continue working. In other words, the recognition of an external authority could be a factor legitimizing the choice of the form of therapy, even in a situation of its negative evaluation.

The recommendations of psychotherapeutic societies had the opposite effect – their indication by people who noticed the disadvantages of online therapy was associated with a lower probability of its use. This may mean that therapists who identify strongly with environmental standards and at the same time are critical of online therapy perceive the recommendations of the societies as insufficient or unconvincing. In this context, it was not an external imperative, but personal clinical judgment and the level of trust in environmental guidelines that could be decisive. Thus, professional recommendations – unlike government recommendations – could act as a selective filter, reinforcing the decision not to introduce online therapy in conditions of negative assessment of its quality.

Qualitative themes suggested that institutional guidance often “tips the balance” when drawbacks are salient. The quantitative mediation results corroborate this pattern: government recommendations increased adoption despite perceived disadvantages, whereas professional-society recommendations co-occurred with continued skepticism and lower adoption. Emotional factors discussed by participants (fear, fatigue) did not statistically mediate decisions, indicating that, in this sample and time-window, institutional context—rather than affect—filtered the impact of perceived drawbacks on behavior.

In conclusion, this research study showed that therapists would see both the advantages of (91.52%) and defects (87.10%) of online therapy. These results provide the basis for continuing research and developing standards and guidelines that could support psychotherapists in the implementation of their work in online therapy, so that this increasingly popular and common form of work is a satisfactory and safe place for therapeutic meetings. Analyzing the decision-making process of psychotherapists in the field of the decision to use online therapy, it has been shown that the specific advantages and disadvantages noticed by psychotherapists are not directly related to the decision to use online therapy. On the other hand, government recommendations and recommendations of psychotherapeutic societies are mediating factors in the relationship between noticing the disadvantages of online therapy and using this form of work. These seemingly similar institutional factors interact in a different way, and while government recommendations, despite psychotherapists’ internal doubts, increase the compliance of behavior with social expectations, environmental recommendations seem to strengthen psychotherapists’ skepticism in the context of professional standards.

### 4.4 Limitations and future research

**Figure 3:**
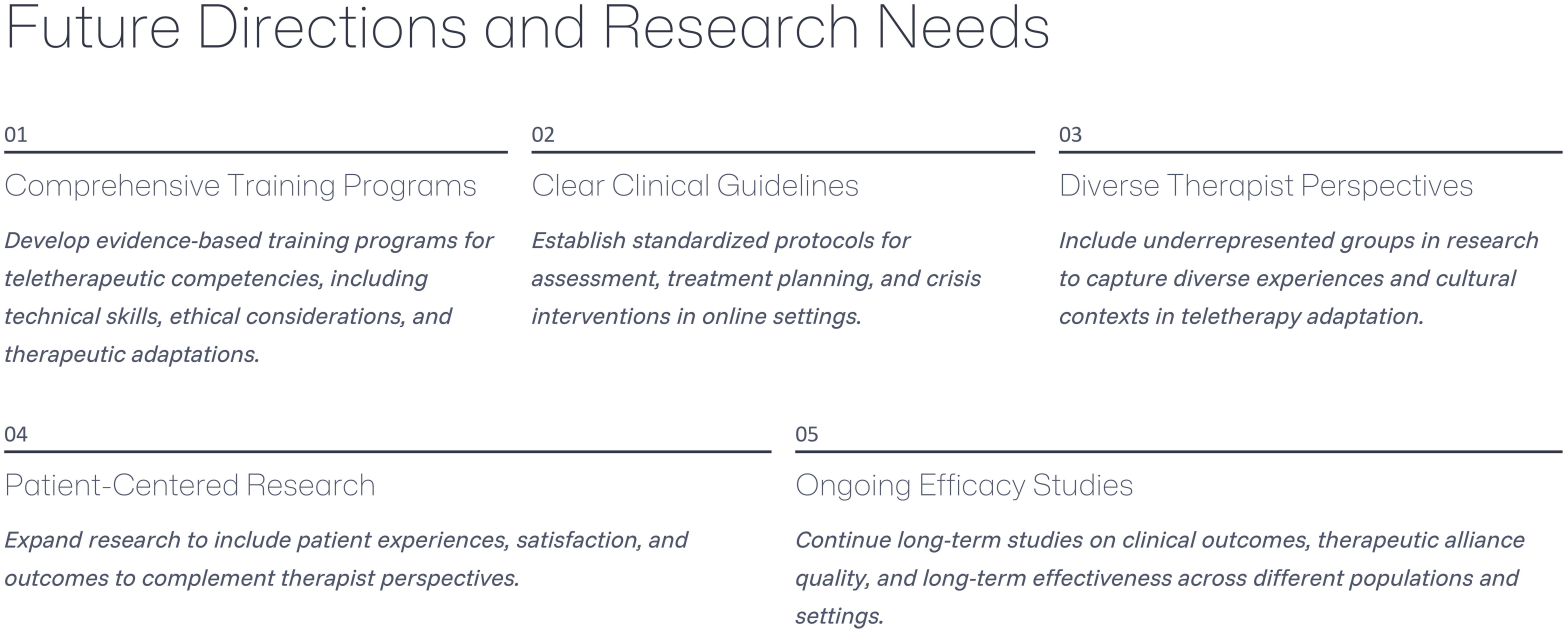
Proposals for Future Research Directions in Online Psychotherapy.

The study is not free from restrictions. This study is exploratory in nature and is based on a voluntary, non-random sample of psychotherapists. Despite a large group of psychotherapists from four European countries, the results, due to the selection of the research sample, do not constitute a representative sample—women as well as practitioners working in large cities and private practices are overrepresented—and participation required individual initiative, which entails a risk of selection bias. Consequently, the results cannot be fully generalized to the entire population of psychotherapists, and future studies should include groups that remain underrepresented, i.e. male therapists, and those working in smaller towns and villages. In addition, the study group is not equal in terms of people using and not using remote therapy. This point is limited to the interpretation and generalization of the results obtained, but at the same time it is important information about the structure of psychotherapists’ work - we assume that it may reflect the actual structure, showing the ratio of people who undertook and did not undertake online work during the pandemic. This is an important point for further analysis (see **Figure 3**).

The survey was conducted during a specific time period of the pandemic. In connection with the international sample, it is worth noting that different countries were in different phases of the pandemic and government restrictions. Also, some psychotherapy and professional societies have already issued some recommendations and recommendations for online therapy, while others have not yet. In addition, in terms of therapists’ experience in remote work, it can be expected that the beginning of the pandemic was associated with less experience in online therapy than the end of the pandemic period, in which many therapists had already gained experience and were increasingly confident in using this format of meetings. Therefore, we can expect that a study conducted at a different time during the pandemic could give slightly different results. It is therefore important to continue research on online therapy, as it does not lose its relevance, and even contradictions, this form is an increasingly common and accessible alternative to therapeutic meetings.

In addition, among the potential mediators of the relationship between the assessment of the advantages and disadvantages of online therapy and decisions about its use, psychological and epidemiological-institutional variables were focused on. These variables do not exhaust the entire complexity of factors that can mediate the studied relationship. It seems reasonable to expand this group to include other variables related to the professional factors of psychotherapists, as well as external factors, e.g. related to the patient and his or her assessment of the advantages and disadvantages of online therapy. The patient’s perspective would allow psychotherapists to relate the benefits and limitations of online therapy to their assessment of this form of work.

The results of the mediation analyses should be interpreted associatively rather than causally. The applied models reflect a hypothetical theoretical sequence (perception of advantages/disadvantages → emotional/institutional factors → decision to conduct online therapy); however, the cross-sectional design does not allow confirmation of temporal precedence and does not rule out circularity (mutual influences X↔M↔Y) or reverse causality (e.g., that the decision to work online already taken modifies, in line with moderation models, the retrospective assessment of its disadvantages). Our results are consistent with such an approach: emotional mediations turned out to be non-significant, and in the “disadvantages → decision” path, small, opposing indirect effects of an institutional nature emerged (government recommendations – positive; professional association recommendations – negative). This indicates that contextual factors may rather condition the strength of the relationship between the perception of disadvantages and the decision (a moderational perspective) than create an unambiguous causal chain. Accordingly, we limit causal language (“mediation”) in favor of formulations describing indirect statistical associations and emphasize the need for longitudinal or experimental designs to determine the direction of effects.

Additionally, it should be noted that mediation inferences relied on bootstrap CIs (advantages models: 2,000 resamples; disadvantages models: 500 resamples); although adequate, future work should use larger and balanced samples with harmonized resampling to increase stability and comparability of indirect effects.

Nevertheless, despite these limitations, the study makes a significant contribution to the understanding of the advantages and limitations of online therapy perceived by psychotherapists and the factors directly and indirectly related to the decision to use it. Although the study concerned the pandemic period, it seems that online therapy has become a permanent part of the job offer of psychotherapists, and with technological progress, we expect a lot of development, research and practical, in this area. Subsequent studies (see **Figure 3**) should examine in detail the advantages and possible limitations of remote therapy, taking into account the specific specificity of different theoretical approaches, as well as both therapist and patient perspectives. This will allow you to create specific recommendations and guidelines for therapists to respond to their specific doubts and needs as much as possible.

## Data Availability

The datasets presented in this article are not readily available because the study was conducted by working psychotherapists and not making the data set publicly available is related to ethical considerations and preventing a situation in which any of the study participants could be identified. Requests to access the datasets should be directed to emiliapsycholog@gmail.com.
